# Stability of peripheral blood immune markers in patients with asthma

**DOI:** 10.1186/s13223-019-0343-4

**Published:** 2019-04-29

**Authors:** Nami Shrestha Palikhe, Ana-Maria Bosonea, Cheryl Laratta, Vivek Dipak Gandhi, Drew Nahirney, Angela Hillaby, Miranda Bowen, Mohit Bhutani, Irvin Mayers, Lisa Cameron, Harissios Vliagoftis

**Affiliations:** 1 Division of Pulmonary Medicine, Department of Medicine, Heritage Medical Research Centre, University of Alberta, Room#550A, Edmonton, AB T6G2S2 Canada; 2 Alberta Respiratory Center, University of Alberta, Edmonton, AB Canada; 3 Department of Pathology, Schulich School of Medicine & Dentistry, Western University, Dental Sciences Building, Rm 4037, London, ON N6A5C1 Canada

**Keywords:** Biomarkers, Immune cells, Peripheral blood, Asthma, Stability, CRTh2, PAR-2

## Abstract

**Background:**

Asthma is a complex disease with variable course. Efforts to identify biomarkers to predict asthma severity, the course of disease and response to treatment have not been very successful so far. We have previous suggested that PAR-2 and CRTh2 expression on specific peripheral blood cell subtypes may be biomarkers of asthma severity. We reasoned that parameters that remain stable when asthma symptoms are controlled would be the most appropriate to evaluate for their utility to predict loss of asthma control and/or severity of the disease.

**Methods:**

Nineteen stable asthmatics were recruited from the University of Alberta Asthma clinic and followed in clinic every 3 months for a total of 4 visits. Patients had spirometry and completed the ACQ questionnaire in every visit. Blood was drawn in every visit and analyzed for a number of immune parameters by flow cytometry. These parameters included PAR-2 and CRTh2 expression on monocyte subgroups and T lymphocytes respectively, as well as numbers of eosinophils, innate lymphoid type-2 cells (ILC2) and dendritic cells. Within person stability of immune and physiological parameters was calculated using the intraclass correlation (ICC) using R version 3.4.0.

**Results:**

FEV_1_ (% predicted), FEV_1_/FVC ratio, ACQ5 and ACQ7 did not differ significantly over the 4 visits, as would be expected for patients with stable asthma. Peripheral blood eosinophil numbers by Kimura stain and by flow cytometry showed ICC scores of 0.44 and 0.52 respectively, indicating moderate stability. The % of ILC2 cells in peripheral blood also showed moderate stability [ICC score of 0.45 (0.14–0.67)]. The stability for all other immune parameters was poor.

**Conclusion:**

Among the peripheral blood immune parameters we studied, only numbers of eosinophils and ILC2 in peripheral blood were moderately stable over a year in stable asthmatics. Further studies are required to understand the reasons for the variability of the other cell types.

## Background

Asthma is a complex disease with multiple phenotypes [1]. The lack of reliable biomarkers to predict frequency and severity of exacerbations or response to new therapeutics makes it difficult to treat appropriately the most vulnerable of asthmatics. The use of induced sputum eosinophils to adjust therapy has been validated, but even the stability of this phenotype over time has been questioned [2]. In the era of biologic drug development, biomarkers are needed to facilitate identification of the patient populations best suited to receive these expensive therapeutics [3].

There has been a recent interest in the stability of proposed biomarkers [4, 5]. Since asthma is a variable and episodic illness with symptoms that are associated with asthma control and/or the presence of exacerbations, individual biomarkers aiming to identify patients with poor asthma control would need to be stable during the stable phase of the disease. It is reasonable to assume that the more stable a biomarker is during periods of stable disease, the higher its utility in predicting asthma exacerbations. In asthma, however, studies have addressed the stability of phenotypes [2, 6], but not that of individual biomarkers.

Chemoattractant receptor-homologues molecule expressed on Th2 cells (CRTh2) is a receptor for PGD_2_ and there has been a lot of interest recently on its role in asthma and the efficacy of CRTh2 antagonists in asthma treatment [7]. CRTh2 is expressed by Th2 cells [8], eosinophils and basophils [9] as well as innate lymphoid type 2 cells (ILC2) [10], all cells that play important roles in asthma pathogenesis [11]. Our previous findings suggest that CD4^+^CRTh2^+^ T cells numbers are increased in patients with severe asthma compared to mild/moderate disease and may be increased primarily in patients with frequent asthma exacerbations [12]. Protease-Activated Receptor-2 (PAR-2) belongs to a family of 7-transmembrane G protein-coupled receptors activated by serine proteases [13] and is expressed in both structural and inflammatory/immune cells in the airways and other tissues. Human peripheral blood monocytes express PAR-2 and release pro-inflammatory cytokines, including IL-6, IL-8, and IL-1β, following PAR-2-mediated activation [14]. We have also shown that patients with severe asthma had increased PAR-2 expression on CD14^++^CD16^+^ (intermediate) monocytes in the peripheral blood compared to those with mild/moderate disease.

Blood eosinophil count is a clinically accessible biomarker for airway diseases [15], now also used as a decision point for treatment of patients with asthma with certain biologics. ILC2 cells are lymphoid cells that lack antigen recognition receptors and produce type 2 cytokines when activated by alarmins produced primarily by airway epithelial cells. They are considered one of the most important cell types in polarizing the immune response towards a Th2 phenotype [16], and have been implicated in the development of allergic lung inflammation [17]. Dendritic cells (DC), the main antigen presenting cells, play a role in allergic diseases not only through antigen presentation, but also as mediators of inflammation [18]. In addition, the numbers of plasmacytoid dendritic cells (pDC) are increased in patients’ sputum during asthma exacerbations and have been correlated with disease severity [19].

The goal of our study was to validate the potential utility of peripheral blood immune parameters associated with the cells discussed above as biomarkers that could predict asthma exacerbations. To do this, we first studied their stability over time in subjects with stable disease. Current and future biologics for asthma and other chronic inflammatory diseases are targeting receptors on immune cells, such as eosinophils, ILC2 and/or dendritic cells (DC), and/or ligands for these receptors. For this reason, we also analyzed stability of eosinophil, ILC2 and DC numbers in peripheral blood as well as stability of other peripheral blood immune parameters in the same subjects. We found that physiologic measures were more stable than cellular measures over a 1-year period.

## Methods

### Subjects

Twenty-two subjects 18–68 years of age with a clinical diagnosis of asthma by a respirologist, which reported no exacerbations for the year prior to recruitment, were recruited from the University of Alberta Asthma Clinic. Three subjects withdrew from the study and 19 completed all 4 visits required by the study. Out of these three subjects, which are not included in the presented data, two withdrew consent after visit 1 and one subject was diagnosed with melanoma before completion and was excluded from further evaluation and analysis. Ethical approval was given by the institutional review board (approval number Pro00001784). Informed consent was obtained from all participants.

Demographics (age and sex), body mass index (BMI), the presence of atopy and serum IgE levels were recorded from the health care record at the time of recruitment. Atopy was determined by positive skin test (at least 3 mm wheal) to 1 or more of a panel of 12 aero-allergens common in the geographic area of the study (timothy grass, birch, poplar, cedar, cat, dog, *D. pteronyssinus*, *D. farinae*, alternaria, aspergillus, hormodendrum, and penicillium).

Each subject attended 4 clinic visits in 3-month intervals. They were also asked to call the clinic if they experienced any significant worsening of their symptoms (exacerbation) in between the visits and to be prepared to come to the clinic for evaluation at those times. Forced expiratory volume in 1 s (FEV_1_), force vital capacity (FVC) and asthma control questionnaires (ACQ) were collected during each visit. Both ACQ5 (nocturnal awakening, morning symptoms, activity limitation, shortness of breath, wheeze) and ACQ7 (ACQ5 questions, plus number of puffs of short acting bronchodilators used each day and FEV_1_ score) were calculated. ACQ7 [20] and ACQ5 [21] questionnaires have been validated. Asthma exacerbations between visits were recorded.

Venous blood was drawn during every visit and immune cells markers were studied in whole blood by flow cytometry. Those were “percentage (%) of eosinophils in peripheral blood (PB)” (by Kimura and flow cytometry), “% of Innate lymphoid type 2 (ILC2) in PB”, “% of CD4^+^CRTh2^+^T cells in PB” and “% of CD4^+^ T cells expressing CRTh2”, “% CD14^++^CD16^+^ (intermediate) monocytes in PB”, “% CD14^++^CD16^−^ (classical) monocytes in PB”, and “% of CD14^++^CD16^+^PAR-2^+^ cells in PB”, “% of CD14^++^CD16^+^ monocytes expressing PAR-2”, “% of myeloid DC (mDC) in PB”, “% of plasmacytoid DC (pDC) in PB”, “% of mDC expressing FcεRIα” and “% of pDC expressing FcεRIα”.

### Profiling of peripheral blood immune cells by flow cytometry

#### Flow cytometry

Peripheral venous blood was collected in sodium heparin tubes (BD Bioscience, Mississauga, ON, Canada) and staining was performed at room temperature (RT) using 100 µl of whole blood.

To block non-specific binding of antibodies Fc blocker (Miltenyi Biotec, Auburn, CA, USA), mouse IgG (50 µg; Invitrogen, Burlington, ON, Canada) and rat IgG (50 µg; Invitrogen) were added to whole blood and incubated for 20 min at RT. Whole blood was then incubated with biotinylated anti-CRTh2 (clone BM16; Miltenyi Biotec) or biotinylated anti-PAR-2 antibodies (Santa Cruz Biotec, Mississauga, ON, Canada) for 30 min at RT. Isotype control antibodies for CRTh2 (rat IgG2a; AbD Serotech, Raleigh, NC, USA) or PAR-2 (mouse IgG1 k; Santa Cruz) were added to other tubes under the same conditions as for primary antibodies.

Red blood cell lysis buffer (BD Bioscience) was then added for 15 min and samples vortexed (10 s) before centrifugation. Cells were centrifuged at 300×*g* (5 min), and washed with 2 ml of PBS (Sigma, St Louis, USA) containing 0.5% BSA, 0.1% NaN3, 3% FBS. For detection of CRTh2^+^ cells and PAR-2^+^ cells, streptavidin-APC (200 ng/µl) was added (eBioscience, San Deigo, CA, USA). Multi-colour flow cytometry was performed using also the following antibodies or isotype controls for cell identification: anti-CD4 (Clone 1F6; Serotec, Oxford, UK), anti-CCR3 (Clone 5E8; BD Bioscience), anti-CD3 (Clone HIT3a; BD Bioscience), anti-CD14 (Clone 61D3; eBioscience), anti-CD16 (Clone 3G8;BD Bioscience), Lin 1 [anti-CD3, anti-CD14, anti-CD16, anti-CD19, anti-CD20, anti-CD56] (BD Bioscience), anti-CD127 (Clone HIL-7R-M21; BD Bioscience), anti-FcεR1 (Clone AER-37 [CRA1]; eBioscience), anti-CD11c (Clone B-ly6; BD Bioscience), anti-CD123 (Clone 6H6; eBioscience), anti-HLA-DR (Clone G46-6; BD Bioscience). BD CompBeads Plus (anti-mouse Ig, ĸ (BD Bioscience) were used to establish compensation corrections for spectral overlap for any combination of fluorochrome-labeled antibodies. Cells and compensation bead tubes were incubated in the dark (30 min), washed with 2 ml of PBS, centrifuged (300×*g*, 5 min) and re-suspended in 250 µl of 2% paraformaldehyde (Sigma Aldrich).

Flow cytometry data were collected on BD LSR Fortessa (BD, CA, USA) using FACS Diva software and gates set in accordance with the profiles of the isotype control and/or negative control beads. Results were analyzed using FlowJo^®^ (TreeStar, Ashland OR, USA).

#### Gating strategy for flow cytometry analysis

**Fig. 1:**
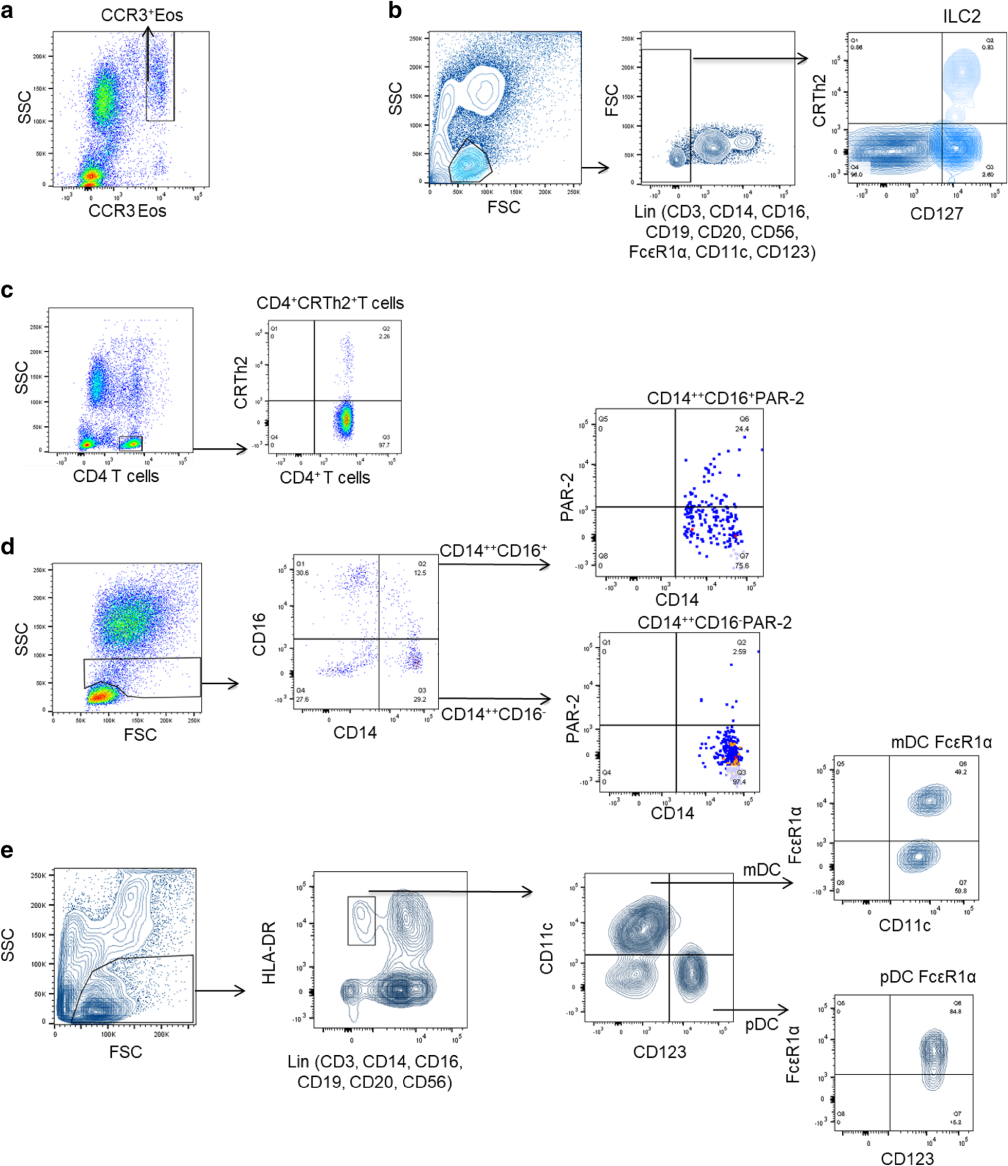
Gating Strategy for flowcytometry analysis of peripheral blood immune parameters

Figure 1a shows the gating strategy for eosinophils that were identified as high side scatter (SSC) and high CCR3 expressing cells (SSC^high^CCR3^high^). Innate lymphoid cells type 2 (ILC2) were identified within the lymphocytes region of an SSC/FSC scatter plot as Lin^negative^ (CD3, CD14, CD16, CD19, CD20, CD56, FcεR1α, CD11c, CD123) cells expressing CRTh2 and CD127 (Fig. 1b). Figure 1c shows the gating strategy for CD4 T cells identified as low SSC high CD4 expressing cells (SSC^low^CD4^high^). This method of identifying CD4^+^ T cells was confirmed with double staining with CD3, which showed the selected population to be over 99% positive for CD3 [12]. Therefore we did not use CD3 staining to identify Th2 cells in this study. The % of CRTh2^+^ CD4 cells was calculated with reference to CRTh2 isotype control. The gating strategy for monocyte subsets was the following (Fig. 1d): cells within the monocyte gate (an area above the lymphocyte region in SSC/FSC scatter plots) were further analyzed for CD14 expression (to identify monocytes) and expression of CD16 to separate CD14^++^CD16^−^ (classical) from CD14^++^CD16^+^ (intermediate) and CD14^+^CD16^+^ (non-classical monocytes) [22]. The classical and intermediate subsets were then analyzed for PAR-2 expression separately. Figure 1e shows the gating strategy for DC. Cells within the lymphocyte and monocyte regions in SSC/FSC scatter plots were identified as DC if they were HLA-DR^positive^Lin^negative^ (Lin in this case included antibodies for: CD3, CD14, CD16, CD19, CD20, CD56). DC were further classified by their expression of CD11c to identify mDC and CD123 to identify pDC. Both subpopulations of DC were further analyzed for their expression of FcεR1α.

### Statistical analysis

Intraclass Correlation coefficient (ICC) was calculated assuming the following model,

$${\text{y}}_{\text{ij}} = \upmu + {\text{u}}_{\text{i}} + \upvarepsilon_{\text{ij}}$$

where y_ij_ represents the outcome value for ith (i = 1, 2, …, 76) patient at jth (j = 1, 2, 3, 4) time period, µ is the overall intercept, u_i_ is the patient-specific random effect and ε_ij_ is the error term. As standard in Linear Mixed Model (LMM), random effect and error are assumed to follow normal distribution: u_i_ ~ N(0, σ_u_^2^) and ε_ij_ ~ N(0, σ_ε_^2^). The ICC is defined as,

$${\text{ICC}} = \sigma_{\text{u}}^{ 2} /\left( {\sigma_{\text{u}}^{ 2} + \sigma_{\varepsilon }^{ 2} } \right).$$

To calculate ICC, FEV_1_ (% predicted), FEV_1_/FVC, ACQ5, ACQ7 and % of CD4^+^CRTh2^+^T cells in peripheral blood were analyzed on original scale. Other variables were transformed to make their distributions normal.

Correlations between various immune parameters and ACQ scores or FEV_1_ were analyzed using Spearman’s or Pearson’s correlation depending on whether the parameters had normal distribution or not respectively. All analyses were performed using R version 3.4.0.

## Results

Nineteen stable asthmatics (no reported asthma exacerbations during the year before recruitment) were recruited from the University of Alberta Asthma clinic after giving informed consent and followed every 3 months for a total of 4 visits. The demographic characteristics of the recruited subjects are shown in Table 1. The mean age of study subjects was 45 years and 63.2% of them were female. Most of the study subjects (93.3%) were atopic (as shown by at least one positive skin test against common environmental aeroallergens). Only three patients had a history of smoking (15.8%) and none of them were current smokers. Patients underwent spirometry, completed the ACQ questionnaire and gave blood for analysis of immune parameters by flow cytometry at every visit. No subject reported or had documented asthma exacerbations during the period of the study.

**Table 1:** Demographic and clinical characteristics of study subjects

	n = 19
Age (y) (Min–Max)	45 (26–64)
Female (%)	12 (63.2%)
BMI (kg/m^2^) (Min–Max)	31.2 (18.1–48.4)
Atopy (%)*	15 (93.3%)
Log transformed IgE (kU/L)* (Min–Max)	1.9 (0.8–3.0)
History of Smoking (%)	3 (15.8%)
Current Smoking (%)	0 (0.0%)

Data was calculated as mean (Min–Max) for continuous variable and percentage for categorical variables

*BMI* body mass index, *IgE* immunoglobulin E, *Min* minimum, *Max* maximum

* n=16 for these parameters; missing data for the other subjects

**Fig. 2:**
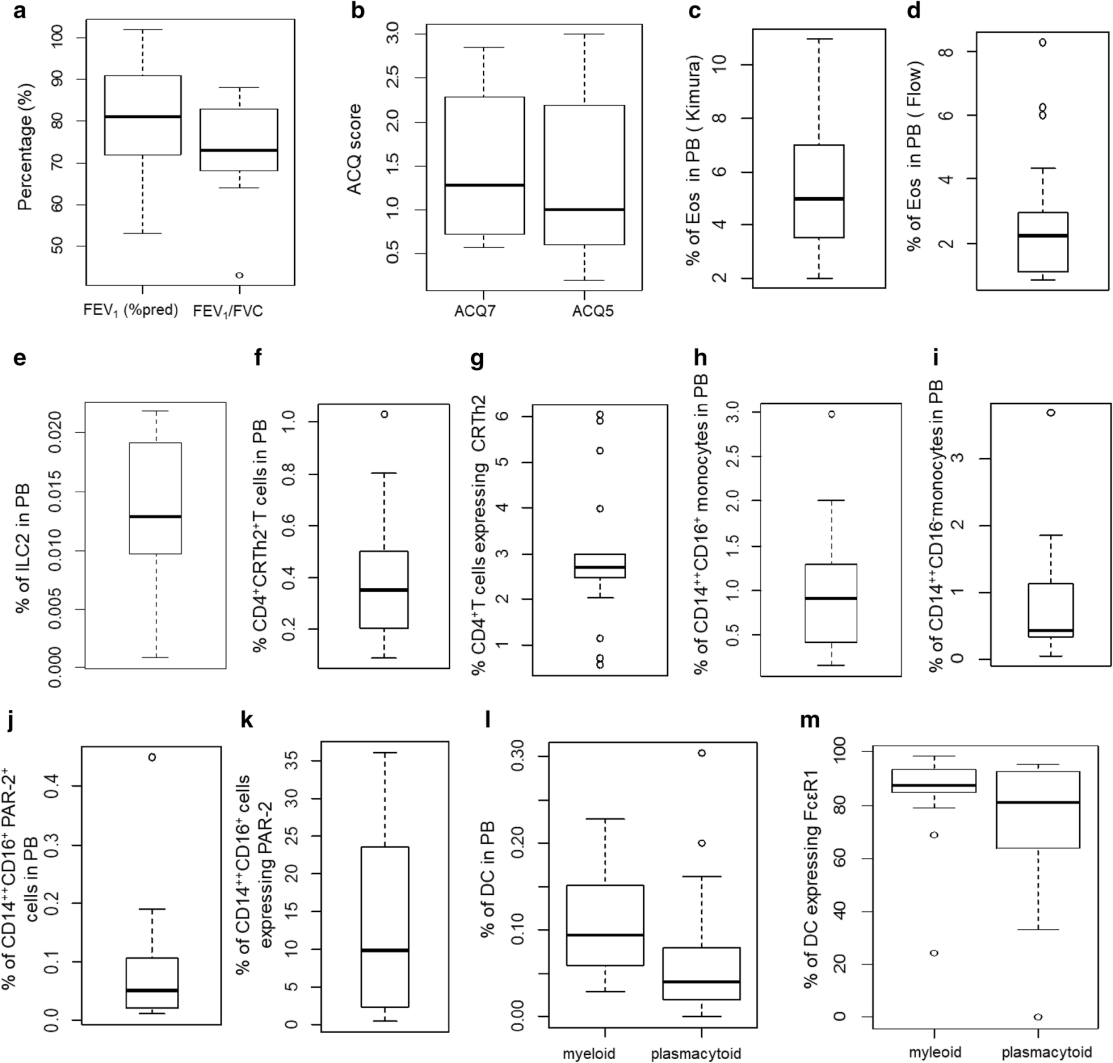
Boxplots of physiological [**a** FEV_1_ (% predicted) and FEV_1_/FVC) and **b** ACQ score (ACQ7 and ACQ5)] and blood immune parameters [**c** % of Eosinophils in PB (Kimura), **d** % of Eosinophils in PB (Flow), **e** % of ILC2 in PB, **f** % of CD4^+^CRTh2^+^T cells in PB, **g** % of CD4^+^T cells expressing CRTh2, **h** % of CD14^++^CD16^+^ monocytes in PB, **i** % of CD14^++^D16^−^ monocytes in PB, **j** % of CD14^++^CD16^+^PAR-2^+^ cells in PB, **k** % of CD14^++^CD16^+^ cells expressing PAR-2, **l** % of DC in PB (myeloid and plasmacytoid), **m** % of DC expressing FcεR1(myeloid and plasmacytoid)] for the whole population during the first visit (recruitment visit)

Figure 2 shows boxplots of all the physiological and peripheral blood immune parameters evaluated at the first visit during recruitment (Fig. 2).

Stability of all these physiological and immune parameters tested was evaluated by Intraclass Correlation coefficient (ICC) and the results are shown in Table 2. FEV_1_ (% predicted), FEV_1_/FVC, ACQ5 and ACQ7 were stable over the 4 visits, as would be expected for patients with stable asthma. Peripheral blood eosinophil numbers evaluated by Kimura stain or by flow cytometry showed ICC scores of 0.44 and 0.52 respectively, indicating moderate stability, similar to what has been previously shown for induced sputum eosinophil counts [2]. “% of ILC2 cells in peripheral blood” showed an ICC score of 0.45 (0.14–0.67), indicating moderate stability. The stability for all other immune parameters was poor (Table 2—all subjects). Furthermore, ICC scores were similar for all biomarkers whether we analyzed the whole population (n = 19), or only female subjects (n = 12) (Table 2).

**Table 2:** Intraclass correlation coefficients (95% CI) by physiological and blood biomarker

	All subjects (n = 19)	Females (n = 12)
Physiological biomarkers		
FEV_1_ (% predicted)	0.90 (0.78–0.95)	0.92 (0.76–0.96)
FEV_1_/FVC	0.79 (0.57–0.89)	0.80 (0.49–0.91)
ACQ5	0.68 (0.44–0.82)	0.72 (0.40–0.86)
ACQ7	0.75 (0.52–0.87)	0.78 (0.46–0.91)
Blood biomarkers		
Eosinophils		
% Eosinophils in PB (Kimura staining)	0.44 (0.10–0.67)	0.50 (0.10–0.76)
% Eosinophils in PB (flow cytometry)	0.52 (0.24–0.71)	0.43 (0.06–0.68)
Innate lymphoid cells (ILC2)		
% ILC2 in PB	0.45 (0.14–0.67)	0.58 (0.08–0.75)
T cell subsets		
% CD4^+^CRTh2^+^T cells in PB	0.17 (0.00–0.40)	0.24 (0.00–0.52)
% of CD4^+^ T cells expressing CRTh2	0.31 (0.04–0.53)	0.35 (0.00–0.62)
Monocyte subsets		
% CD14^++^CD16^+^ (intermediate) monocytes in PB	0.06 (0.00–0.28)	0.00 (0.00–1.00)
% CD14^++^CD16^−^ (classical) monocytes in PB	0.18 (0.00–0.41)	0.06 (0.00–0.38)
% CD14^++^CD16^+^PAR-2^+^ cells in PB	0.24 (0.00–0.44)	0.21 (0.00–0.50)
% CD14^++^CD16^+^ cells expressing PAR-2	0.09 (0.00–0.31)	0.22 (0.00–0.52)
Dendritic cell (DC) subsets		
% myeloid DC (mDC) in PB	0.19 (0.00–0.43)	0.18 (0.00–0.47)
% plasmacytoid DC (pDC) in PB	0.30 (0.04–0.53)	0.30 (0.00–0.60)
% mDC expressing FcεR1α	0.10 (0.00–0.32)	0.12 (0.00–0.41)
% pDC expressing FcεR1α	0.32 (0.04–0.55)	0.20 (0.00–0.51)

*FEV*_*1*_ forced expiratory volume in 1 s, *FVC* forced vital capacity, *ACQ5 and ACQ7* asthma control questionnaire based on 5 or 7 questions, *PB* peripheral blood

We then attempted subgroup analysis to test whether we can identify predictors of biomarker stability. We hypothesized that lower variability of ACQ between the 4 visits would indicate more stable disease. We calculated the difference between the highest and lowest ACQ for each of the participants and then grouped them according to the median value of this difference. There was no significant difference in ICC scores for any of the immune parameters studied between subjects with low (less than the median) and high (higher than the median) ACQ variability (data not shown). In a similar analysis we divided the subjects in terms of having lung function better or worse than the median. Subjects with “FEV_1_ (% predicted)” over the median of the population, those with less severe obstruction, had better asthma control compared to those with “FEV_1_ (% predicted)” below the median (ACQ7 1.056 ± 0.113 vs. 1.906 ± 0.171, p < 0.001). Those subjects also had more stable ILC2 numbers (ICC: 0.52 (0.01–0.77) vs. 0.17 (0.00–0.56)), as well as higher “% ILC2 cells in peripheral blood” (0.015 ± 0.014% vs. 0.008 ± 0.015%, p = 0.003). All other parameters we evaluated showed no differences in stability between subjects with high and low FEV_1_ (% predicted) (data not shown).

**Fig. 3:**
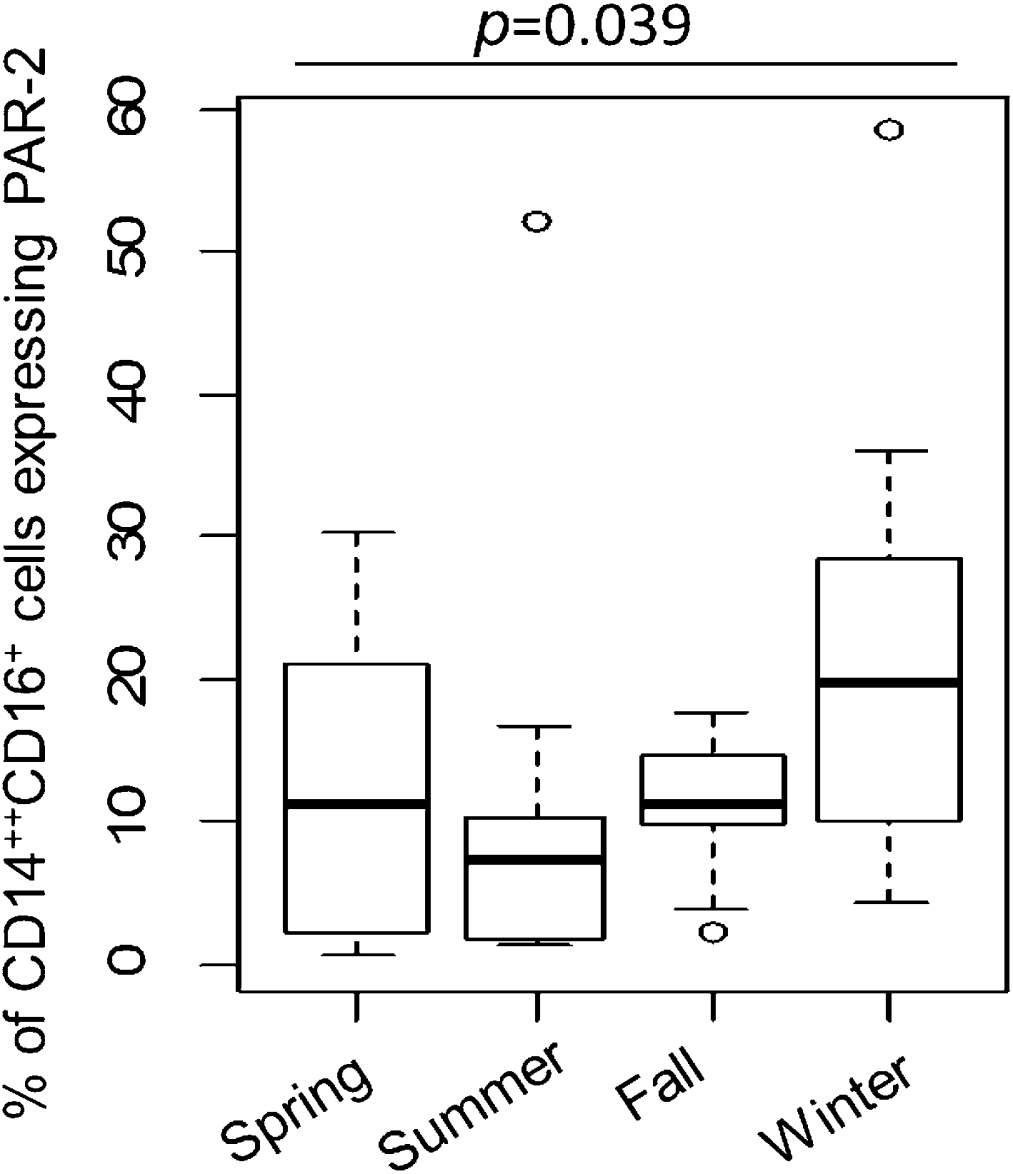
Boxplots of “% of CD14^++^CD16^+^ cells expressing PAR-2” according to four seasons (spring, summer, fall, winter) (N = 14 for each season)

To address whether immune cell markers vary seasonally, we selected the 14 patients that had their 4 visits falling within all 4 seasons [spring (March 20 to June 20), summer (June 21 to Sept 21), fall or autumn (Sept 22 to Dec 20) and winter (Dec21st to March 19)]. With the exception of % CD14^++^CD16^+^ cells expressing PAR-2, the mean values of the physiological and immune parameters we measured were not significantly different between the 4 seasons, indicating that these parameters do not show seasonal variation in our population. The mean values of % CD14^++^CD16^+^ cells expressing PAR-2 were different among the four seasons (*p *= 0.039), with the value being the highest during winter while the other seasons had comparable numbers (Fig. 3).

**Fig. 4:**
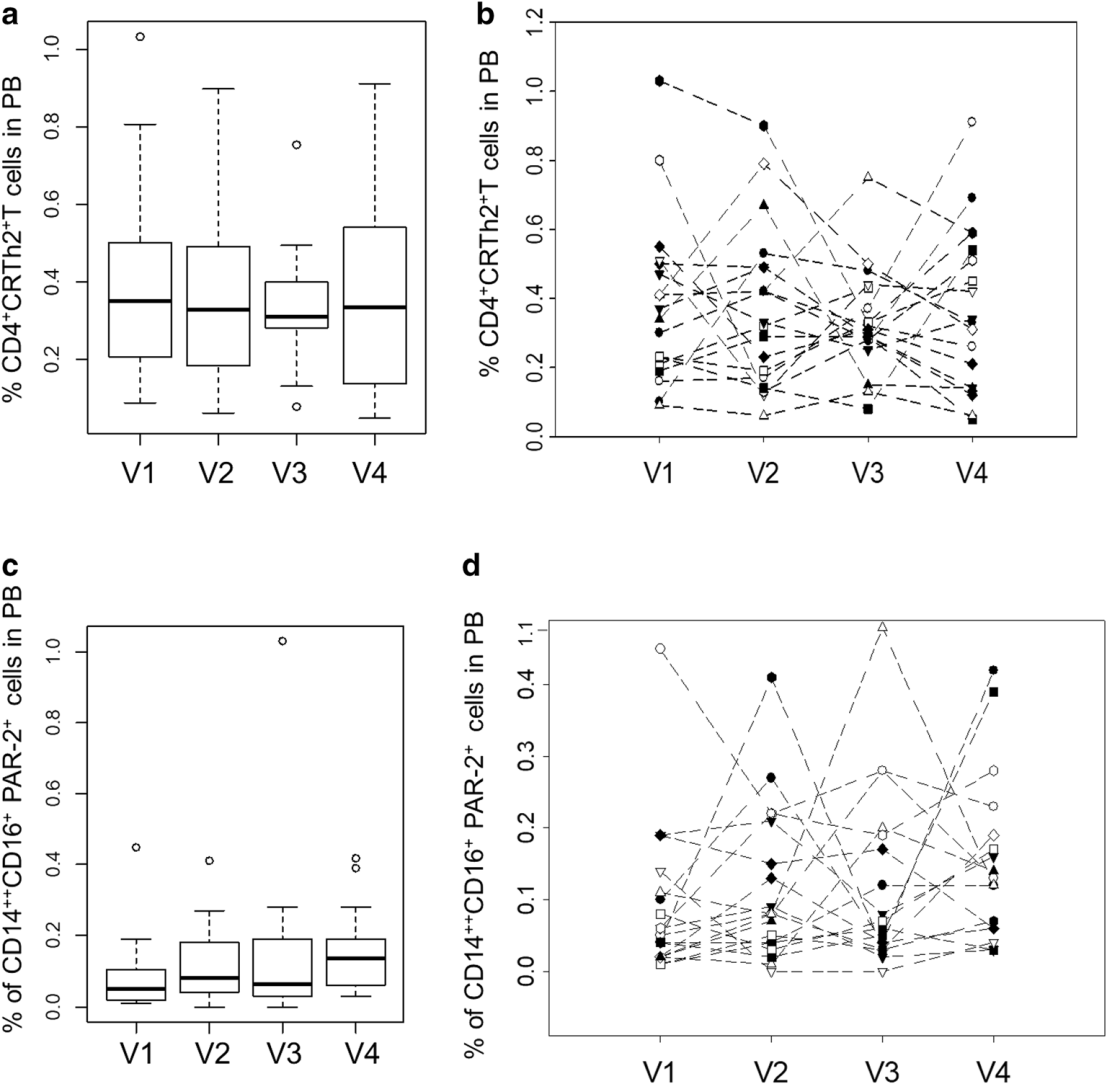
Boxplots of “% CD4^+^CRTh2^+^T cells” in PB (**a**) and “% of CD14^++^CD16^+^PAR-2^+^” cells in PB (**c**) results from the whole study population for each one of the four visits. Line graph of “% CD4^+^CRTh2^+^T cells” in PB (**b**) and % of “CD14^++^CD16^+^PAR-2^+^” cells in PB for each patient in four visits (**d**)

In previous publications we suggested that “% of CD4^+^CRTh2^+^T cells” [12] and “% of CD14^++^CD16^+^PAR-2^+^ monocytes” [22] may be good biomarkers of asthma severity. However, here both these parameters showed poor stability in patients with stable asthma. We showed that the population had a median of 0.35% and 0.05% for these two parameters during the first visit (Fig. 2f, j). We then compared this data from visit 1 with that of visits 2–4 (Fig. 4a, c). There was no statistical difference for the results of the whole population between the four visits for either parameter, although individual patients showed significant changes in both parameters across the four visits (Fig. 4b, d).

## Discussion

In this manuscript we evaluated the stability of flow cytometric evaluation of a number of peripheral blood immune parameters in stable asthmatics and showed that the majority of these parameters show poor stability over 1 year. Two among these parameters, the percentage of PAR-2 expressing intermediate monocytes and the percentage of Th2 cells in peripheral blood, which we have previously shown to be elevated in blood of severe asthmatics [12, 22].

The reasons for the variability of the various immune parameters we studied are not clear. The variability did not correlate with changes in pulmonary function or ACQ during the same period (data not shown), indicating that reasons other than asthma control may be responsible. Part of the variability may depend on the characteristics of our flow cytometry-based assay. For our assays, we stained fresh whole blood cells followed by fixation and analysis by flow cytometry. To discuss one possibility of how the use of fresh cells may affect the results, mechanical stress affects PAR-2 expression on monocytes [14], and therefore may alter observed PAR-2 expression. An assay using fixed cells would be preferable clinically, since this approach would allow for cells to be fixed immediately after collection from patients and subsequent analysis be done at a convenient time in a central facility.

We also did not take into consideration diurnal [23] or seasonal variation [24] of the measured parameters, which may have affected stability. Most subjects were seen in the clinic in the morning, but we cannot rule out the possibility that time of sample collection had an effect on the observed variability. Regarding seasonal variation, it is interesting that the cellular composition of blood including monocyte numbers is increased during winter [24]. In our case, % CD14^++^CD16^+^ cells (intermediate monocytes) expressing PAR-2 also showed the highest values during winter while the three other seasons had comparable numbers. The reasons for this increase in % CD14^++^CD16^+^ cells expressing PAR-2 during winter is not clear. This change might be the result of higher indoor levels of allergens in winter due to the cold weather and lack of window opening, on viral infections being more frequent in winter, or on other factors. Further studies in larger cohorts are needed to clarify this issue.

It is interesting that from all the immune parameters we analyzed only % ILC2 and % eosinophils in peripheral blood reached moderate stability. The importance of eosinophils in asthma and asthma exacerbations is well studied. Blood eosinophil numbers have also been used as a biomarker for response of severe asthmatics to newer biologics [25]. In this study, we report that blood eosinophil counts have an ICC of 0.52 in this cohort of 19 patients and these values are comparable with ICC scores for blood eosinophils in COPD [26] and for induced sputum eosinophils in asthmatics [27]. The reason for the moderate variability of eosinophil numbers is not clear. However, it is known that blood eosinophil counts can by influenced by many factors, including diurnal variation, exercise and the use of corticosteroids [28].

ILC2 have been recently proposed as major players in asthma and their numbers decrease after initiation of treatment [29]. Although Tian et al. showed that peripheral blood ILC2 is negatively correlated with FEV_1_ [30], we observed higher percentage of peripheral blood ILC2 among those asthmatics who had less severe airway obstruction. The reason for this is not clear at this point. Further studies would be required to understand the association between ILC2 cells in peripheral blood and airway obstruction and/or asthma severity. ILC2 cells and eosinophils are both cells characterized by CRTh2 expression. The stability of these two CRTh2-expressing cell types during periods of stable disease raises the possibility that total blood CRTh2 expression may be a viable biomarker for asthma exacerbations. Indeed, we have previously reported that CRTh2 mRNA levels may be a biomarker of severe asthma [12].

We have previously suggested that “% of CD4^+^CRTh2^+^T cells” [12] and “% of CD14^++^CD16^+^PAR-2^+^ monocytes” [22] are biomarkers of asthma severity. The present study showed low stability of these parameters over 1 year in subjects with fairly stable asthma, as shown by spirometry and asthma control questionnaire. To test whether these immune parameters were stable in the population as a whole, we compared these parameters for the whole population (19 subjects) between visits 1, 2, 3 and 4. There was no difference in the values for “% of CD4^+^CRTh2^+^T cells” and “% of CD14^++^CD16^+^PAR-2^+^ monocytes” for the whole population between the 4 visits. This could indicate these two cell populations may be more useful for identifying patients with a severe asthma phenotype, rather than acute changes in disease activity or asthma control. Further studies are needed to better understand the utility of these immune parameters as biomarkers.

For this study we did not evaluate any of the immune parameters directly in the lungs. It is clear that sputum eosinophil count is an excellent biomarker of asthma activity, increases during exacerbations and responds to treatment. Otherwise, there is limited information on the expression of the other immune parameters in the airways. Studies analyzing induced sputum and/or BAL showed that allergen challenge increase the number of ILC2 and Th2 lymphocytes present in the airways [31], and the activation status of these cells. ILC2 expressing CRTh2 were also overrepresented in BAL and induced sputum from children with severe therapy resistant asthma compared to peripheral blood [32], while severe asthmatics also had higher CRTh2 mRNA expression in BAL, compared to patients with mild-moderate asthma and healthy controls [33]. Detailed studies of all immune biomarkers in lung tissue and blood in the same patients would confirm the validity of blood biomarkers, which are easier to obtain in every day practice.

## Conclusion

In summary, while pulmonary function and asthma control were quite stable in this population over 1 year, the majority of peripheral blood immune parameters we analyzed showed poor stability. A previous study has also concluded that clustering patients with asthma according to physiological parameters is more stable longitudinally than using immune markers in sputum [2]. This finding would indicate that there are also other determinants of immune cell number and activation in the blood of patients with asthma in addition to the stability of disease as shown by ACQ values. Further studies are needed to understand the reason for the observed variability of these immune parameters and what this variability may mean for asthma pathophysiology and for our quest to identify biomarkers of asthma severity and/or control.
